# News Items

**Published:** 1895-01

**Authors:** 


### NEWS ITEMS.

THE nineteenth annual meeting of the Pennsylvania State Board of Agriculture and General Farmers' Institute was held on January 23d and 24th at Harrisburg. On the last day it was addressed on "Should Dairy Cows be Inspected, and, if so, Should it be Done by State Authority?" by Geo. Abbott, Philadelphia, Pa.; "Tuberculosis at the State Hospital for the Insane, Norristown, Pa.," by Florence Hull Watson, pathologist of the asylum, Norristown, Pa.; "Tuberculosis," by Dr. D. E. Salmon, Chief of the Bureau of Animal Industry, Washington, D. C.; "Tuberculosis," by Dr. Leonard Pearson, University of Pennsylvania, Philadelphia, Pa, and general discussion of the tuberculosis question.

Bulletin No. 4, of the Montana Agricultural Experiment Station, pertaining to glanders, by W. L. Williams, veterinarian, has just been received. It will bear reading by every veterinarian; and the light form the disease assumes in that high altitude will attract attention. It contains excellent advice to the people of Montana, and all veterinarians will, after reading his quotations of many of those who have given an opinion as to its curability, appreciate his conclusions and advice as to the value of such an attempt.

Much criticism as to the great expense upon the State Treasury of Massachusetts in the work of eradicating tuberculosis is being indulged in by the public press, but we are full sure that if the object is well attained that there will be no more appreciative body of people in the land than those of that State.

The State of New Hampshire is now considering measures necessary to a more complete eradication of tuberculosis, and a large appropriation is asked for from the present Legislature. This legislation is being pressed for by the Board of Cattle Commissioners, which was created four years ago. When animals are killed one-half their appraised value on the basis of good health is allowed. This valuation is not in many instances considered sufficient. She charges much of her tuberculosis to Massachusetts when formerly it was the custom to graze many of the former's cattle on New Hampshire soil. Under the present law 900 herds have been examined, and over 600 head destroyed. Tuberculin has been depended upon the last four months for the detection of the disease.

The last number of the *Sugar Trade Journal* contained the following interesting item:

"Mr. Vivien, professor at the Sugar Academy at Douai, proposes, as one of the means to augment the consumption of sugar in accordance with the development of the production, the use of sugar for the alimentation of cattle, by giving 200 grammes (nearly one-half pound) of sugar per head of cattle, and if given during 200 days of the year to the 41,200,000 cows, horses, hogs, and sheep which exist in France, the French cattle united would eat the enormous quantity of 1,764,000,000 kilos of sugar, or twice our entire production. If the English cattle, numbering about 38,000,000 animals, consumed the 200 grammes of sugar per head for 200 days in the year, England would consume for this purpose 1,500,000 tons. The use of sugar in the alimentation of cattle, even in the country where sugar is cheapest, would offer a limited outlet."

Bulletin No. 44 has been received from the Department of Animal Industry, relating briefly the means of diagnosis, treatment, etc., of swine disease and hog cholera. Considerable importance is placed upon the value of the following prescription as a curative measure when the disease has broken out:

| | lbs. |
|---|---|
| ℞.—Wood charcoal | 1 |
| Sulphur | 1 |
| Sodium chloride | 2 |
| Sodium bicarbonate | 2 |
| Sodium hyposulphite | 2 |
| Sodium sulphate | 1 |
| Antimony sulphide | 1 |

Pulverize and mix thoroughly.

DOSE—A large tablespoonful for each 200 pounds of hogs to be treated. Given in the feed or, drenched, mixed with water when necessary. It is also recommended as a preventive remedy, especially when the disease prevails in a locality.

